# The Ultra-Scientific Non-Practical Writer

**Published:** 1902-08

**Authors:** 


### THE ULTRA-SCIENTIFIC NON-PRACTICAL WRITER.

THE following pertinent reflections are taken from the *St. Louis Medical and Surgical Journal:*

There is a large number of intelligent physicians wondering how it is that there are comparatively so few who need first-class scientific publications, and so many who eagerly buy and read journals which upon their very face others judge to be the veriest rot. This can be very easily explained in this country. The medical journal which permits a subscriber to ask a question concerning the cure of a disease is equally ready to print twenty answers, each of which furnishes what was asked for by the ones who made the interrogation. This is what the rank and file will call practical. What does it matter whether the question is foolish and the answer incorrect? Every one concerned in the transaction is satisfied, and presumably contented. What matters it whether the patient for whose benefit the query and answer were made profits by the maneuvre or not? The journal has shown what its true function is and thereby has earned the lasting gratitude of all of its subscribers, not one of which has failed to profit by the information so freely given. Some there are who are so unappreciative of the benefits conferred by such a method that they go so far as to say that it is merely a case of the blind leading the blind. But he who does not possess a good library, and who lacks that fundamental education which every one should possess, finds refuge in this sanctuary designed for the ignorant and careless. What is the use of studying it when others do it for you? That is the question, and with this excuse they are willing to rust out.

But it is those who deny this condition of affairs who are the principal cause of it. They study and learn much. They do not keep their knowledge to themselves. Far be it from us to accuse them of such selfishness. On the contrary, they scatter their scientific work and contributions broadcast, and thereby earn fame. But their work is a little too scientific for the rank and file. They are ultra-scientific and sacrifice everything on the altar of science. They devote all their energies to the pursuit of the scientific side of a question alone, and lose sight of the fact that the hard work-a-day physician wants something more than mere theories; he hungers for that which can be made useful and turned to some practical account and benefit. In other words, he desires methods of worth and applicability, not theories and hypotheses which have not even been proven. He is perfectly right from his point of view. When he asks for bread he is not satisfied with wind-pudding. He must have something substantial, something which contains nutrition. His mind is not adapted to, nor has it been trained to be satisfied with mere words. It needs something upon which it can lean when fatigued by the practical problems which it is forced to face and grapple with so often; that despair makes it clutch even at a straw. This is the reason that the non-scientific journal which has many prescriptions, good, bad and indifferent, has such a large following and enjoys so much popularity. It is certain to possess a large constituency, and one which will not easily relinquish it. It is a wrong principle to feed babies on lion's marrow when the utmost they can have is pap. The food must be gradually changed until our medical infant can digest the best and the strongest which is furnished to it.

To pursue the subject still further: It is a well-known fact that the most intelligent soon tires of the ultra-scientific. Too much learning is apt to drive any one mad. It is an intellectual diet which finally palls upon the best appetite. It is of disadvantage in that it creates a dislike for that which, whilst not being so scientific, at least appeals to the ingenuity and resources of a trained intellect. Our devourer of ultra-scientific material has adopted for his motto, *aut Cesar aut nullus*. Give him pure science or give him nothing. He has adopted the gross and practical, and leaves such things to those who have debased ideas, and whose finer sensibilities to the purely scientific have been dulled by too much attention to things of the earth earthy. But how can they do otherwise, when they know nothing of transcendentalism? It is well for him who has been educated in such things to become ultra-scientific, and it is meet that he should cultivate this gift, but it is his duty to confine its expression to those of his own habit of thought. All of which leads us to the question: Can there possibly be some means by which both extremes can be brought into a harmonious rapport? Can matters not be so adjusted as to combine into one well-balanced whole, and thus be equally pleasant and useful to all? Is is necessary to go to extremes, when a means of more utility and of greater pleasure to all can be adopted? We are not in the reforming business, we are merely giving suggestions, which, if acted upon, would without doubt lead to results, and such results as would eventually be of benefit to mankind. The death of the hyperpractical medical journal would not necessarily follow; on the contrary, it would be improved just for the very reason that it is eminently practical to adopt adaptation to environments.

We are entirely in favor of scientific journals, but they must, of necessity, be limited to those capable of understanding their contents and of being contributors. There is no necessity whatever for that which is brutally subservient to that which poses as the practical. There is a necessity, and a great one, for the journal which is just scientific enough, and has enough of the practical in it to be of real worth to him who is in search of help. None is so learned, or so well endowed mentally, that he cannot profit by the work and thoughts of others. There is none so poor but what he can make his contribution, present his scientific obole to his brethren.

We still invite our readers to contribute, whether it be scientific or practical. Whichever one it may be, some one else will be found who will fill the lacuna, if such there be. We must all work together and each one contribute, and thus create an interesting literature.

